# Decoding key cell sub-populations and molecular alterations in glioblastoma at recurrence by single-cell analysis

**DOI:** 10.1186/s40478-023-01613-x

**Published:** 2023-07-31

**Authors:** Xin Wang, Qian Sun, Weiwen Wang, Baohui Liu, Ying Gu, Liang Chen

**Affiliations:** 1 RNA Institute, Hubei Key Laboratory of Cell Homeostasis, College of Life Sciences, Department of Neurosurgery, Renmin Hospital of Wuhan University, Wuhan University, Wuhan, 430072, China; 2 BGI Research, Hangzhou, 310030 China; 3 China National GeneBank, BGI Research, Shenzhen, 518120 China; 4 BGI Research, Shenzhen, 518083 China; 5 Guangdong Provincial Key Laboratory of Genome Read and Write, BGI Research, Shenzhen, 518083 China; 6 College of Life Sciences, University of Chinese Academy of Sciences, Beijing, 100049 China

**Keywords:** Glioblastoma, Recurrence, scRNA-seq, MAFK, NFATC4, Tumor microenvironment

## Abstract

Glioblastoma (GBM) is the most frequent malignant brain tumor, the relapse of which is unavoidable following standard treatment. However, the effective treatment for recurrent GBM is lacking, necessitating the understanding of key mechanisms driving tumor recurrence and the identification of new targets for intervention. Here, we integrated single-cell RNA-sequencing data spanning 36 patient-matched primary and recurrent GBM (pGBM and rGBM) specimens, with 6 longitudinal GBM spatial transcriptomics to explore molecular alterations at recurrence, with each cell type characterized in parallel. Genes involved in extracellular matrix (ECM) organization are preferentially enriched in rGBM cells, and MAFK is highlighted as a potential regulator. Notably, we uncover a unique subpopulation of GBM cells that is much less detected in pGBM and highly expresses ECM and mesenchyme related genes, suggesting it may contribute to the molecular transition of rGBM. Further regulatory network analysis reveals that transcription factors, such as NFATC4 and activator protein 1 members, may function as hub regulators. All non-tumor cells alter their specific sets of genes as well and certain subgroups of myeloid cells appear to be physically associated with the mesenchyme-like GBM subpopulation. Altogether, our study provides new insights into the molecular understanding of GBM relapse and candidate targets for rGBM treatment.

**Supplementary Information:**

The online version contains supplementary material available at 10.1186/s40478-023-01613-x.

## Introduction

Glioblastoma (GBM) is the most prevalent brain tumor. Current therapeutic strategies include surgical resection, radiotherapy, and temozolomide chemotherapy [1]. Although primary GBM (pGBM) patients receive intensive treatments, the relapse is inevitably and the median survival is only 15 months [1–3]. On the other hand, there is no effective treatment for recurrent GBM (rGBM) [4, 5], partially due to the incomplete mechanistic understanding of GBM relapse and lack of promising candidate targets for rGBM therapy [6].

GBM cells are highly heterogeneous, leading to therapy resistance and poor prognosis. The Cancer Genome Atlas (TCGA) classified GBM patients into four subtypes (proneural, neural, classical, and mesenchymal) based on bulk tissue data, highlighting the inter-patient heterogeneity and indicating specific treatments are required for each subtype [7]. Subsequent studies further reported intra-patient heterogeneity by showing multiple TCGA subtypes of cells within individual tumors, which was further demonstrated by single cell sequencing [8–13]. In parallel, a recent landmark work proposed a four-state model (neural-progenitor-like, oligodendrocyte-progenitor-like, astrocyte-like, and mesenchymal-like) for all GBM patients tested, while the ratio of each state was variable [13]. Given its importance in determining each tumor’s characteristics, a full understanding of the rGBM heterogeneity at both cellular and molecular levels becomes necessary to search for combination of targets to cover all tumor cells.

Notably, the relative proportion of GBM cells in each state does not remain constant in the same patients, but rather undergoes dynamic changes as disease progresses or in response to treatment [13–16]. Specifically, it has been shown the rGBM underwent a therapy-related mesenchymal transition based on bulk genomics [17–23], which was later demonstrated by deconvolution of bulk RNA-seq [24] and single cell transcriptomic studies [14–16]. Mechanistically, the single cell investigation into patient-matched pGBM and rGBM revealed certain key regulators, including activator protein 1 (AP-1), that mediating the mesenchymal transition [15]. Furthermore, quiescent cancer stem cells were identified as a persistent population and drive GBM recurrence [25]. Thus, the high-resolution comparison of longitudinal GBM specimens that cover different stages/situations would help capture the dynamic nature as GBM progresses, and key mediators.

To fully dissect the cellular and molecular transitions of GBM, including both tumor and non-tumor cells, we analyzed scRNA-seq data of patient-matched primary and recurrent specimens from a recently published study [15]. By analyzing both commonly and uniquely altered genes for pGMB and rGBM cells, we showed that extracellular matrix (ECM) organization is enriched in GBM cells at recurrence, which could be represented by a set of signature genes. Importantly, we identified an emerged subpopulation of rGBM cells that highly expresses ECM-related genes and shows evident mesenchymal transition, possibly via several transcription regulators, such as NFATC4. On the other hand, we characterized the heterogeneity of myeloid cells, certain subgroups of which were spatially associated with mesenchymal-like subpopulation of tumor cells. Finally, we constructed an online interface for the exploration of the analyzed dataset (https://db.cngb.org/cdcp/visualization?project=CNP0004174).

## Materials and methods

### Data collection

Published data were collected for this study. In detail, scRNA-seq data of patient-paired primary and recurrent specimens were retrieved from the Gene Expression Omnibus repository (https://www.ncbi.nlm.nih.gov/geo/), and the accession code is GSE174554 [15]. Specifically, the specimens used in this study were filtered by two criteria: with patient-paired primary and recurrent data available; with the detected cell number above 1000. The Glioma Longitudinal Analysis (GLASS) RNA-seq datasets were retrieved from https://www.synapse.org [17]. The Chinese Glioma Genome Atlas (CGGA) RNA-seq datasets were downloaded from GlioVis data portal (http://gliovis.bioinfo.cnio.es) [26, 27]. The spatial transcriptomic (ST) data of longitudinal GBM samples were retrieved from the Github website (https://github.com/adithyakan/reconvolving_gbm) [28].

The signatures used in this research were collected from published studies and online databases. Specifically, the anatomic features were retrieved from the publication of Ivy GBM atlas project (Ivy GAP) [29], the GBM stem cell signature (GS) and proliferating signature (P) were obtained from the publication of Xuanhua et al. [25]. Additionally, the differential peaks enriched in rGBM cells and the motifs over-represented in rGBM-specific peaks were obtained from the publication of Lin et al. [15], the intrinsically expressed genes in GBM were acquired from the publication of Qianghu et al. [22]. The GBM diagnostic markers were mainly collected from the fifth edition of the WHO Classification of Tumors of the Central Nervous System [30].

### Preprocessing of scRNA-seq data

The scRNA-seq data were processed by Seurat package (version 4.0.5) [31]. Specifically, the expression matrix of each sample was read in by Read10X function, and each Seurat object was created by CreateSeuratObject function. Then, the poor quality data were filtered out by setting the parameters min.cells as 3 and min.features as 200. The datasets from different samples were integrated with Seurat, and then the integrated object was normalized by NormalizeData function and scaled by ScaleData function. Subsequently, 3000 variable genes were identified and the top 30 PCAs were calculated for the following embedding and clustering. Finally, the clustering result was visualized by Dimplot function.

### Cell-type identification

In the section of cell-type identification, the canonical cell-type markers were collected and the expression of each marker was mapped onto the t-distributed stochastic neighbor embedding (t-SNE) plot by FeaturePlot function. Meanwhile, the violin plots were used to visualize the expression by ggplot2 (version 3.3.5). In detail, the used markers include *EGFR*, *PTPRZ1* (GBM cell); *APOE*, *ADGRV1* (astrocyte); *MBP*, *CTNNA3* (oligodendrocyte); *APBB1IP*, *CD74* (myeloid); *SYT1*, *MYT1L* (neuron); *CD247*, *CD96* (T cell).

### Copy number variation (CNV) analysis

The inferCNV package (version 1.8.1) [9] was used to deduce the CNV for each cell. In this process, the myeloid and oligodendrocyte were regarded as the normal references for CNV calculation. The CreateInfercnvObject function was called to create the inferCNV object, and then the function run was used for CNV inferring. The crucial parameters in this process include cutoff = 0.1 and HMM = T.

### Subpopulation analysis of GBM cells

To subcluster GBM cells, we first selected GBM cells across longitudinal samples and then split them into different Seurat objects by SplitObject function. Subsequently, we normalized and identified variable features for each object. Then, we prepared for the integration by SelectIntegrationFeatures and FindIntegrationAnchors functions. The different Seurat objects were integrated by IntegrateData function. The following clustering and embedding steps were the same as mentioned above.

### Identification of rGBM gene signature (rGBM GS)

In the process of rGBM GS prediction, the differential expressed genes (DEGs) were first identified by FindAllMarkers function in Seurat, and the parameters were set as follows: min.pct = 0.1, logfc.threshold = 0.25, and return.thresh = 0.05. To obtain the credible DEGs upregulated in rGBM samples, p_val < 0.01, avg_log2FC > 0.3, and pct.1 > 0.25 were set. Then, the DEGs were filtered by intersecting with the GBM intrinsically expressed genes [22] and the selected genes were further submitted to STRING [32] to obtain the gene module. The genes in the module were identified as rGBM GS.

### Gene ontology (GO) analysis

The GO analysis in this study was performed by clusterProfiler package (version 4.0.5) [33]. In detail, the enrichGO function was called and the parameters were set as follows: OrgDb = org.Hs.eg.db, keyType = SYMBOL, ont = type, qvalueCutoff = 0.05, and pvalueCutoff = 0.05. Finally, the GO terms (biological processes) were obtained and visualized by ggplot2 (version 3.3.5).

### Protein–protein interaction (PPI) network analysis

To construct the PPI network, the selected DEGs were submitted to STRING website (https://cn.string-db.org/) [32] in multiple-proteins column and the organism of *Homo sapiens* was selected. Then, we chose the matched proteins and ran the PPI analysis to generate the PPI network.

### Deconvolution analysis

To infer the cellular proportions from bulk RNA-seq data, the GLASS and CGGA datasets were collected and further deconvoluted by CIBERSORTx [17, 26, 34]. For all the two runs, the scRNA-seq matrix analyzed in this study, which spanning normal cell types and GBM cell subpopulations, was used as the input single-cell reference for CIBERSORTx to obtain the signature matrix. Subsequently, the obtained signature matrix and the bulk RNA-seq matrix were used for the inference of cell proportions. S-mode was set to correct the batch effect and 100 was set for permutation in significance analysis. After the cellular proportions were obtained, the GBM cell subpopulations were selected to calculate the relative proportion.

### Pseudo-time trajectory analysis

The pseudo-time trajectory for GBM cells was constructed by monocle package (version 2.18.0) [35]. First, the monocle object was created by newCellDataSet function, the functions of estimateSizeFactors and estimateDispersions were called for preparation. Then, the highly dispersion genes were calculated by dispersionTable function and further selected by following settings: mean_expression >= 0.1 and dispersion_empirical >= 1 * dispersion_fit. The cells along the trajectory were arranged by orderCells function and visualized by plot_cell_trajectory function.

### Prediction of transcription factors (TFs)

To predict the TFs for GBM cells in primary and recurrent samples respectively, the expression matrixes of GBM cells in each sample were submitted to RABIT (http://rabit.dfci.harvard.edu/) [36]. Then, the predicted TF lists were obtained, as well as their regulatory activity scores. Specifically, the TFs with positive score were considered to be activated in the corresponding samples.

### Gene module analysis

To refine the gene modules of myeloid in GBM, the Python packages Scanpy [37] and Hotspot [38] were used. First, the myeloid cells were selected and further filtered by Scanpy according to the following settings: n_top = 20, min_genes = 500, and min_cells = 20. Then, the create_knn_graph function in Hotspot was applied to compute the K-nearest-neighbors graph with n_neighbors = 300, and compute_autocorrelations function was used to compute autocorrelations for each gene. Finally, we retained the top 1000 significantly correlated genes and grouped them into modules.

### Survival analysis

The relationship of gene with prognosis was evaluated by GEPIA [39] and GlioVis [27]. Specifically, the expression of the indicated gene was retrieved and the median value was settled as the boundary to classify patients into the high or low expression groups. The log-rank test was calculated to examine whether there is a significance difference in prognosis between the two groups. The results were visualized by Kaplan–Meier (KM) plots.

### Statistical analysis

All statistical analyses (Student's t-test, Wilcoxon rank-sum test, and log-rank test) were performed by R, and the results were considered statistically significant if the *p* value < 0.05.

## Results

### The single-cell profiling on longitudinal GBM specimens

**Fig. 1:**
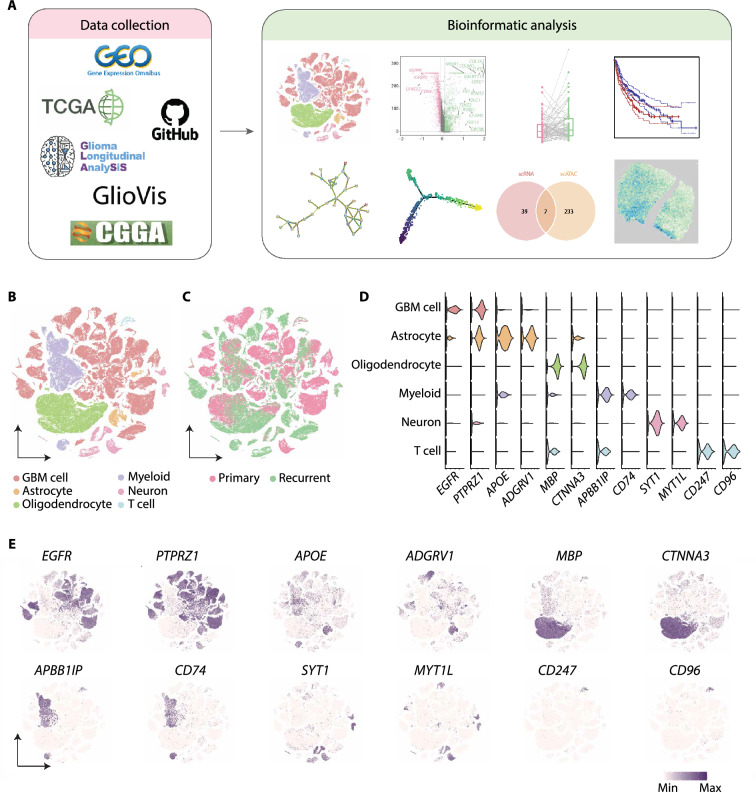
The single-cell transcriptomic profiling of paired pGBM and rGBM specimens. **A** Schematic diagram of the experimental workflow. **B** t-SNE plot showing single cells recovered from pGBM and rGBM samples, labeled by cell type. **C** t-SNE plot colored by pGBM and rGBM samples. **D** Violin diagram showing the expression of canonical cell-type markers in scRNA-seq. **E** t-SNE plot showing the expression of canonical cell-type markers. The purple color represents the higher expression

To profile the molecular alterations of GBM at recurrence, we collected the scRNA-seq data of patient-paired primary and recurrent specimens from a recently published study [15] and 18 pairs of high-quality datasets were selected (see methods) (Fig. 1A). After quality control, 118,031 cells were obtained and classified into 22 clusters according to the expression similarity of highly variable genes (Additional file 1: Fig. S1A-E). We annotated the cell clusters based on the expression of canonical cell-type markers (Fig. 1B–E; Additional file 6: Table S1). Finally, six cell types were identified, including GBM cell, astrocyte, oligodendrocyte, myeloid, neuron, and T cell, in accordance with the previous study [15].

To validate the accuracy of GBM cell assignment, we deduced the copy number variation (CNV) for all cells by inferCNV [9] (Additional file 1: Fig. S1F), and distinguished the malignant cells from others according to the CNV changes at chromosomes 7 and 10 (+7/−10) [9, 40]. The result showed that cells with the typical CNV features were highly overlapped with the tumor cell clusters identified by canonical cell-type markers, therefore confirming the accuracy of the GBM cell assignment we performed (Fig. 1B; Additional file 1: Fig. S1F).

In accordance to previous findings, GBM cells from different specimens were grouped into separate clusters, suggesting the inter-patient heterogeneity, as well as the longitudinal heterogeneity of the same patient (Fig. 1B, C; Additional file 1: Fig. S1D, E). Altogether, the single cell transcriptome profiled here for patient-paired samples provides a molecular basis for further dissection of cell state transition in GBM.

### An overview of molecular alterations of GBM cells at recurrence

A systematic characterization of molecular transitions in GBM under therapy will not only enhance our mechanistic comprehension for tumor relapse and drug resistance, but also represent an urgent need to search for new therapeutic targets for relapsed patients [41]. To this end, we first extracted the DEGs in pGBM and rGBM cells respectively, taking astrocytes as the control (Additional file 1: Fig. S2A; Additional file 7: Table S2). 2,722 and 2,879 upregulated genes were identified in pGBM and rGBM cells respectively. Notably, 2,301 (85% and 80%, respectively) upregulated genes were shared by both groups, consistent with the previous study and implying that rGBM largely reserved the transcriptome features as pGBM [42]. Of note, several well-known genes involved in glioma progression were abundantly expressed in both groups of tumor cells (Additional file 1: Fig. S2A), including cell growth-related (*EGFR*, *NRG3*, and *APOE*) and migration-related genes (*VEGFA* and *ASTN2*). These tumor-related features were also highlighted in the functional enrichment analysis (Additional file 1: Fig. S2B). Other highlighted terms were GTP-related, such as Ras protein signal transduction, which was also involved in oncogenic transformation and tumorigenesis [43–45].

**Fig. 2:**
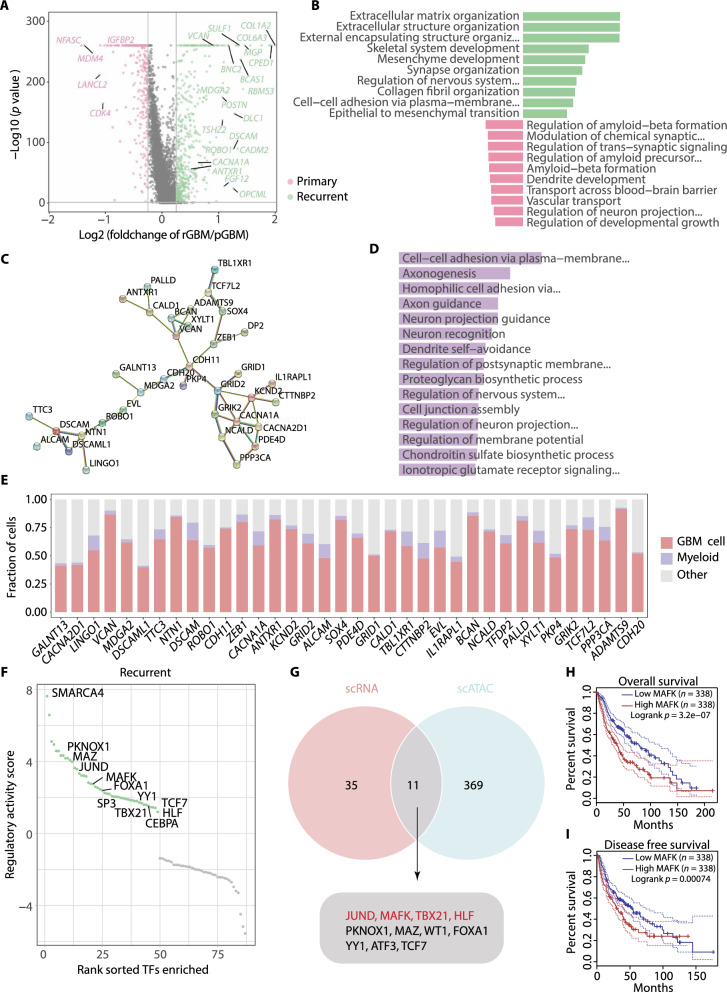
Integration analysis reveals the molecular alteration of GBM cells at recurrence. **A** Scatter plot showing the DEGs between pGBM and rGBM cells. **B** Bar plot showing the enriched GO terms (biological processes) for DEGs in Fig. 2A, the colors correspond to Fig. 2A. **C** Network showing the PPI in rGBM GS identified by STRING [32]. **D** Bar plot of the enriched GO terms for rGBM GS. **E** Stacked bar plot showing the proportion of indicated cells expressing rGBM GS genes. **F** Scatter plot showing the regulatory activity scores for deduced TFs activated in GBM cells from recurrent samples by RABIT [36]. **G** Venn diagram showing the intersection of TFs deduced by scRNA-seq data and differential peaks from scATAC-seq data [15]. **H** and **I** Overall survival (**H**) and disease-free survival (**I**) curves of glioma patients stratified by MAFK expression using GEPIA [39]

There were groups of genes upregulated separately in primary or recurrent tumor cells as well (Additional file 1: Fig. S2A). To gain more information about the difference between two groups of specimens, we directly compared the transcriptome of pGBM and rGBM cells (Fig. 2A; Additional file 8: Table S3). 343 genes were significantly upregulated in rGBM cells, such as *GALNT13*, *ROBO1*, and *ANTRXN1*. These genes were enriched in the functions of ECM, mesenchyme development, and cell-cell adhesion (Fig. 2B; Additional file 9: Table S4), suggesting that relapsed GBM cells gain more capability in mediating ECM reorganization and undergo a transition towards mesenchymal state, consistent with the features of rGBM reported in previous studies [14–16, 24, 42]. In contrast, genes involved in synapse organization, dendrite development, and regulation of nervous system development were either up- or down-regulated in rGBM compared with pGBM, implying both groups of tumor cells gain certain neuronal phenotype, but with distinct gene sets (Fig. 2B).

To extract the gene signature that represents the rGBM feature, we took the intrinsically expressed genes in GBM and their PPI interactions into consideration, a similar strategy as previously described [46]. Specifically, we filtered the DEGs with the 11,529 GBM intrinsically expressed genes [22] and submitted the selected genes to the STRING tool [32]. A rGBM-specific 36-gene-module with PPI was acquired, namely rGBM GS (Fig. 2C; Additional file 1: Fig. S2C). Functional analysis indicates rGBM GS genes were highly enriched in cell adhesion and synapse organization pathways (Fig. 2D; Additional file 10: Table S5), in accordance with the overall DEG features (Fig. 2B). Moreover, rGBM GS genes were primarily expressed in GBM cells compared with other non-tumor cells, further suggesting rGBM GS could represents the inherent molecular feature of rGBM cells (Fig. 2E).

### Dissection of potential regulators underlying the transition of rGBM cells

Given the evident transcriptomic difference between pGBM and rGBM cells, we were prompted to explore the underlying transcription regulators that preferentially function in rGBM cells. We retrieved the expression matrixes for each type of GBM cells and submitted them to the RABIT [36], a platform to predict regulators that shape the gene expression. Then, 66 and 49 predicted regulators for pGBM and rGBM cells with positive regulatory activity scores were obtained, respectively (Fig. 2F; Additional file 2: Fig. S2D; Additional file 11: Table S6). Among them, SMARCA4, the top hit in the list for rGBM cells, is involved in cell growth and ECM organization, and is essential for proliferation and migration in diffuse midline glioma [47]. Knockdown of the *SMARCA4* gene in MCF-10A cells has been shown to result in downregulation of ECM genes [48]. JUND, another top hit, belongs to AP-1 family and has been reported to enhance the expression of mesenchymal genes at recurrence (Fig. 2F) [15].

 The single-cell assay for transposase-accessible chromatin with sequencing (scATAC-seq) was recently developed to profile the open-chromatin regions, from which the regulatory information of genes, including the gene activity and potential TFs could be inferred from a given cell population [49, 50]. To further retrieve confident TFs essential for GBM progression, we intersected the rGBM-specific regulator list obtained above with two regulator lists deduced using scATAC-seq data in the previous study, which were obtained from either direct differential motif enrichment test or TF motif search from differential peaks between pGBM and rGBM cells [15]. As the result, 11 and 7 regulators were supported by two sets of data respectively (Fig. 2G; Additional file 2: Fig. S2E), many of them have been reported in mesenchymal signature regulation, such as JUND [15], SP3 [51], and CEBPA [52].

Notably, MAFK was ranked as one of top hits in both lists (Fig. 2G; Additional file 2: Fig. S2E). It belongs to the small MAF family of transcription factors [53]. While it has not been extensively studied in glioma, aberrant expression of MAFK was reported in a previous study of triple-negative breast cancer to promote tumorigenic growth and metastasis with induced EMT phenotype [54], making it a potential regulator for the enhanced mesenchymal feature of rGBM. To examine this possibility, we performed a target gene prediction analysis for MAFK by the ChIP-Atlas tool, according to MAFK binding information [55]. Strikingly, two third of the rGBM GS genes were shown as the MAFK targets, strongly supporting the contribution of MAFK in mediating the acquired features of rGBM (Additional file 12: Table S7). We further performed Kaplan–Meier analysis [39, 56–58], which showed that high expression of MAFK predicted shorter overall and disease-free survival in patients with glioma (Fig. 2H and I; Additional file 2: Fig. S2F). Collectively, these results indicate that MAFK may function as a master regulator in rGBM cells and associates with poor prognosis.

### A GBM subpopulation with high angiogenesis and ECM production emerges at recurrence

**Fig. 3:**
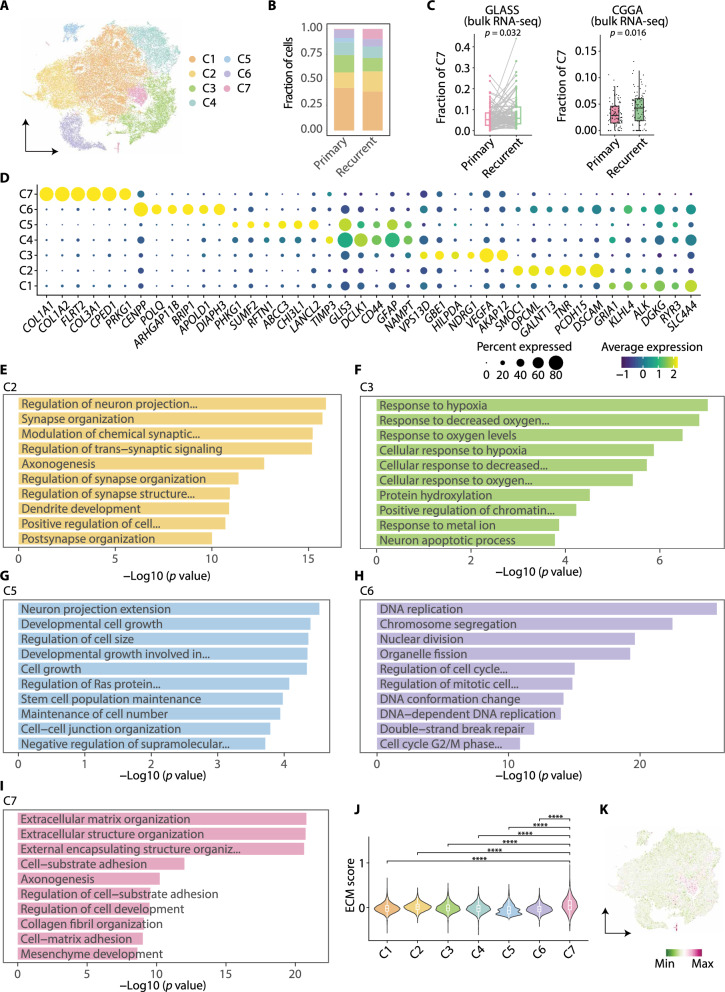
Single-cell transcriptome resolves heterogeneity of GBM cells from pGBM and rGBM samples. **A** t-SNE plot showing subpopulations of GBM cells. **B** Stacked bar chart showing the fraction of GBM cell subpopulations across primary and recurrent samples. **C** Box and ladder plots showing the difference in the deconvolved fractions of C7 between pGBM and rGBM in GLASS (left) and CGGA (right). **D** Dot plot showing the expression of top six DEGs in each GBM cell subpopulation. **E**–**I** Bar plots depicting the enriched GO terms (biological processes) for DEGs in each GBM cell subpopulation. **J** Violin plot showing the expression scores of ECM signature for each GBM cell subpopulation. **K** t-SNE plot showing the expression scores of ECM signature

The transcriptomic difference between pGBM and rGBM cells (Fig. 2A, B) raises an intriguing question as to whether the molecular transition seen in rGBM cells is attributed to the change of the whole population of tumor cells or preferentially a subset of cells, which cannot be fully addressed by bulk sample data [42]. On the other hand, the heterogeneity of rGBM cells has just begun to be studied and how tumor cells at recurrence behave differently is incompletely understood [15, 24, 42, 59]. To tackle the above question and explore the heterogeneity of rGBM, we retrieved the GBM cells across the longitudinal samples and performed clustering analysis according to their transcriptomic similarity. Seven subpopulations were obtained and the proportion of C7 was considerably increased at recurrence, suggesting its potential importance with tumor progression (Fig. 3A, B; Additional file 3: Fig. S3A-E). As the number of patients for the scRNA-seq data analysis was small, we next included additional datasets to examine the existence of C7 and its increase in proportion at recurrence. We performed deconvolution analysis of the bulk RNA-seq data collected from GLASS and CGGA using reference cell-type signatures from the analyzed scRNA-seq [17, 26, 34]. Consistently, the fraction of C7 cells was significantly increased in rGBM in both datasets (Fig. 3C; Additional file 13: Table S8).

We next explored top DEGs and the enriched GO terms to gain functional insights for each GBM cell subpopulation (Fig. 3D–I; Additional file 3: Fig. S3F-G; Additional file 14: Table S9). Several terms were shared by the subpopulations, such as neuron projection related processes displayed in C1, C2, C4, and C5, indicating the process extending from neural cells was a common phenomenon in the majority of GBM cells. Nevertheless, the subpopulations were distinguished by their specific characteristics. In detail, C1, C2, and C4 were featured with GTPase regulation, synapse organization, and axonogenesis, respectively. Hypoxia response (as well as genes such as *VEGFA* and *NDRG1*) was enriched in C3, together with neuron apoptotic process. C5 contains actively expressed genes in cell growth and stem cell population maintenance. C6 was highlighted by proliferation (as well as genes such as *CENPP*) and DNA repair (genes such as *POLQ* and *BRIP1*). Intriguingly, genes involved in ECM organization (genes such as *COL1A1*, *COL1A2*, and *FLRT2*) and the mesenchyme development were highlighted in C7, two features for rGBM cells in total tumor cell comparison above (Fig. 2A, B). To further evaluate the contribution of C7 to the global ECM-related gene expression, we scored the ECM signature for all subpopulations and C7 displayed the highest score compared with others (Fig. 3J, K). Additionally, the AP1 and TNF signaling, which have been reported in the MES transition of GBM [15, 60], were both significantly scored higher in C7 compared with other subpopulations (Additional file 3: Fig. S3H, I). Thus, these findings suggest that a specific subpopulation (C7) emerged at recurrence and may play a significant role in ECM production within rGBM. A possible scenario is that, tumor cells surrounding MVP region in rGBM tend to be activated by microenvironment, such as higher inflammatory signals, and transition to mesenchymal-like state by key pathways/regulators, including AP-1.

The heterogeneity of primary and recurrent GBM cells brings an interesting question as to how various diagnostic markers would be represented in different subpopulations. We therefore visualized the expression of 28 diagnostic markers, either mutated or aberrantly expressed in glioma (Additional file 3: Fig. S3J). Several markers, such as *ATRX* and *EGFR*, were expressed in most of the clusters, while other markers showed subpopulation-specific expression levels, such as *CD34* (blood vessel associated) and *ERG* (EMT associated) in C7, *MKI67* (proliferation associated) and *PARP1* (proliferation associated) in C6, and *RBFOX3* (neuron projection) in C2, which largely corroborated with the molecular features of corresponding subpopulations. Collectively, this result suggests that the expression of canonical diagnostic markers for glioma may likely represent part of the tumor cells in patients.

### Distinct molecular features and potential regulators of GBM subpopulations at recurrence

**Fig. 4:**
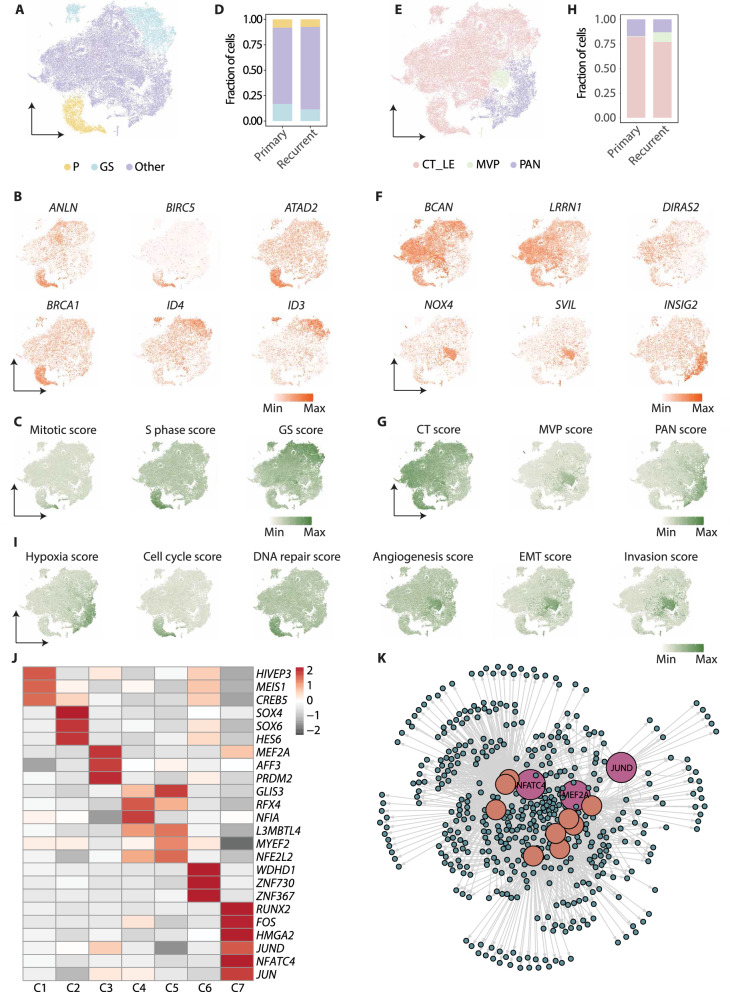
Regulatory network highlights NFATC4 as the hub regulator for rGBM-emerged cell subpopulation. **A** t-SNE plot colored by subgroups classified by P and GS signatures. **B** t-SNE showing the expression of genes from P and GS signatures. **C** t-SNE plot showing the expression scores for mitotic, S phase, and GS signatures. **D** Stacked bar plot showing the proportional composition of P and GS subgroups. **E** t-SNE showing subpopulations characterized by anatomic features profiled by Ivy GAP. **F** t-SNE plot showing the expression of genes from Ivy anatomic features. **G** t-SNE plot showing the expression scores for Ivy anatomic features. **H** Stacked bar plot showing the proportional composition of subgroups assigned by Ivy anatomic features. **I** t-SNE plot showing the expression scores of signatures from CancerSEA. **J** Heatmap showing the TFs for each GBM cell subpopulation deduced from the DEG list. **K** TF regulatory network showing the predicted candidate TFs and target genes in C7

It has been reported that tumor cells with divergent states were present in GBM, including proliferating GBM cells (P cells) and quiescent GBM stem cells (GS cells), and GS cells were proposed as a driver population for the relapse [25]. To facilitate the functional annotation of different cell subpopulations we observed, we classified the GBM cell subpopulations based on the expression of individual marker genes (*ANLN*, *BIRCS*, *ATAD2*, and *BRCA1* for P cells; *ID3* and *ID4* for GS cells) (Fig. 4A, B; Additional file 4: Fig. S4A), and the expression scores of the gene signatures for P and GS cells (Fig. 4C; Additional file 15: Table S10). Strikingly, C6 were assigned to the P cells, C4 and C5 to GS cells, indicating a high consistency of our subclustering strategy with the previous method. While there was a subtle decline of the percentage of GS cells at recurrence, P cell number remained unchanged, suggesting active growth in both pGBM and rGBM (Fig. 4D; Additional file 16: Table S11).

We next defined the tumor cell subpopulations based on the anatomically distinct regions that were dissected and profiled by Ivy GAP. Then, three groups were assigned: the mixture of cellular tumor and leading edge (CT_LE), microvascular proliferation (MVP), and pseudopalisading cells around necrosis (PAN) (Fig. 4E-H; Additional file 4: Fig. S4B) [29]. Most of the subpopulation cells, including C1, C2, and C4-C6, were assigned to CT_LE, indicating a complex and intermingled cell composition of the main tumor mass. C3, which preferentially expressed genes in response to hypoxia and apoptotic process, and received high hypoxia score from CancerSEA [61] (Fig. 4I), fully coincided with PAN marker gene expression (*INSIG2*, *HILPDA*, and *NDRG1*) and PAN signature score (Fig. 4F, G). This result implies that C3 represent tumor cells close to the hypoxia region and thus tend to undergo cell death [42]. Intriguingly, C7, specifically enriched with ECM and mesenchyme features, was classified as MVP based on the marker gene expression (*NOX4*, *SVIL*, and *EDNRA*) and signature score (Fig. 4F, G). This subpopulation was also scored high for angiogenesis, EMT, and invasion signature from CancerSEA [61] (Fig. 4I). To further validate spatial relationship of C7 and MVP regions in GBM, we analyzed the ST data from longitudinal GBM tissues [28]. Strikingly, cells featured with C7 cells tend to cluster together in a restricted area with high score of ECM, MVP, angiogenesis, EMT, and invasion in rGBM (Additional file 4: Fig. S4C). Similar yet often weaker overlaps of these features were also seen in pGBM. These findings suggest an interesting scenario, in which cells belonging to C7, are mainly originated from the area around the blood vessels and express higher levels of ECM-related genes. Such a unique microenvironment would in turn facilitate mesenchymal transition and invasion of tumor cells [42, 62, 63].

Distinct characteristics and functions of rGBM subpopulations imply different transcription regulation. We scanned the DEG list and picked TFs uniquely expressed in each GBM subpopulation (Fig. 4J). For example, the cell cycle related WDHD1 was revealed in C6 [64, 65]. ECM-related RUNX2 [66, 67] was identified in C7, which has been reported to promote EMT [68, 69], drive ovarian cancer chemoresistance [70], and induce hepatocellular carcinoma development [71]. Moreover, NFATC4 was involved in ECM production during cardiac myofibroblast differentiation [72] and liver fibrosis [73]. Other TFs, such as JUND, the members of AP-1 family, was reported to be involved in the mesenchymal transition [15], consistent with the enriched mesenchyme feature of C7 (Fig. 3I and 4I). To resolve the credible regulators in C7 regulation, we performed the TF prediction analysis according to the binding motifs across the DEGs by iRegulon. As shown in the figure, NFATC4 was assigned to be at the hub of the regulation network (Fig. 4K), further indicating its key regulatory role and might be the most potential candidate for C7 targeting.

### Dynamic molecular transition of GBM cells along the differentiation trajectory

**Fig. 5:**
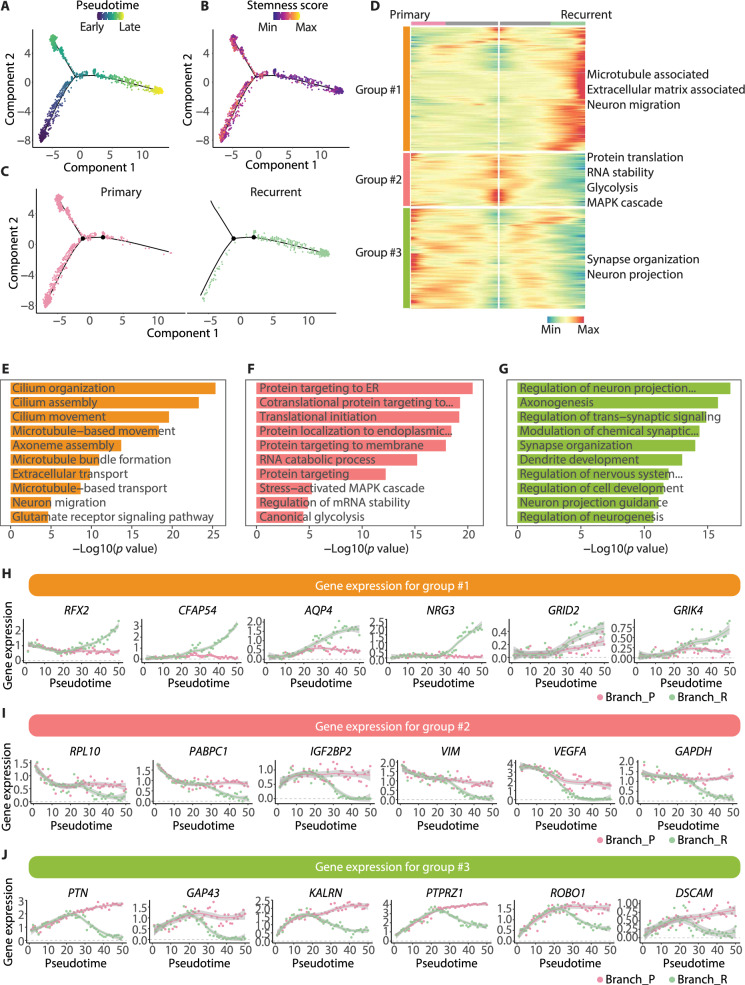
Pseudo-time analysis reveals the distribution difference of GBM cells across the differentiation trajectory. **A** Monocle trajectory for GBM cells from the primary and recurrent samples. The color indicates the pseudo time. **B** Monocle trajectory as in Fig. 5A. The color indicates the expression score for stemness signature. **C** Distribution of GBM cells from the two types of samples on the trajectory. **D** Heatmap depicting the groups of branch-dependent genes according to their expression patterns along the trajectory. **E**–**G** Bar plots showing the GO terms (biological processes) enriched by genes in group #1 (**E**), group #2 (**F**) and group #3 (**G**) from Fig. 5D. **H**–**J** Scatter plots showing the expression of genes in group #1 (**H**), group #2 (**I**) and group #3 (**J**) from Fig. 5D

Data from longitudinal GBM samples provided us with an opportunity to deduce the gene expression transition along with GBM progression. To this end, we subjected the longitudinal GBM cells to the pseudo-time trajectory analysis, the root of which was identified according to the stemness score of the cells (Fig. 5A, B). Markedly, the trajectory was mainly rooted in pGBM cells and two branches arose subsequently, one was distributed only by GBM cells from primary samples (namely branch_P) and the other mainly by recurrent samples (namely branch_R) (Fig. 5C). The lower stemness score of rGBM cells depicted on the trajectory was consistent with their MES transition and decreased proneuronal state at recurrence described previously [14–16, 24].

To further reveal the dynamic alteration of gene expression along the trajectory, we divided the trajectory associated genes into three groups according to their expression patterns (Fig. 5D; Additional file 17: Table S12). Then, three groups were obtained: group #1, genes upregulated along branch_R and little change along branch_P; group #2, genes downregulated along with branch_R and subtle changes along branch_P; group #3, upregulated first and then downregulated along branch_R, but upregulated along branch_P (Fig. 5E–J; Additional file 18: Table S13). Functional analysis revealed that pathways associated with microtube, ECM, and neuron migration were enriched in group #1 (genes such as *RFX1*, *CFAP54,* and *AQP4*), indicating the cell contacts were more active in the evolution trajectory occupied by rGBM cells. In group #2, protein translation, MAPK, and glycolysis were enriched (genes such as *RPL10* and *PABPC1*), implying the discrepancy of basic metabolism along the two branches. Additionally, the synapse organization and neuron projection processes were involved in group #3 (genes such as *PTN* and *GAP43*), indicating though neuronal signals were observed in both types of GBM cells (Fig. 2B), the expression of the underlying molecules is dynamic along the trajectory and exhibits differently between the two branches.

### All non-tumor cells undergo molecular changes at recurrence

As various types of non-tumor cells have been implicated in GBM progression as well [15, 42], we were curious about how non-tumor cells would act in rGBM. To this end, we analyzed the DEGs of each type of non-tumor cells between the two types of samples and performed functional analysis to explore the enriched processes in rGBM (Additional file 5: Fig. S5A-E; Additional file 19: Table S14). Specifically, oligodendrocyte harbored more ability for neuron cell-cell adhesion, glial cell differentiation, and myelination (Additional file 5: Fig. S5A), indicating that more contacts of oligodendrocytes with other cell types were established at recurrence. Additionally, neuron was enriched in synapse organization related processes, implying considerable neuron network re-organization and contact formation occur in rGBM (Additional file 5: Fig. S5B). Astrocyte was featured with RNA metabolism and protein translation, indicating a higher gene expression and protein production activity in astrocyte at recurrence (Additional file 5: Fig. S5C).

In addition, both myeloid and T cell experienced shifted activities related to various types of immune responses (Additional file 5: Fig. S5D, E), indicating an immune environment remodeling at GBM recurrence. The transcriptome transition of myeloid cells has been reported in GBM of MES subtype compared with that of other subtypes or the normal brain, yet its relationship with the MES transition was not fully explored [24]. Thus, we scored the signatures representing myeloid cells in MES subtype of GBM, and visualized the expression scores on ST images. Interestingly, the spots with highly MES-specific myeloid scores located in the C7 niche of rGBM (Additional file 5: Fig. S5F), implying a potential link of myeloid cells in the formation of C7 state in ECM production and MES transition.

**Fig. 6:**
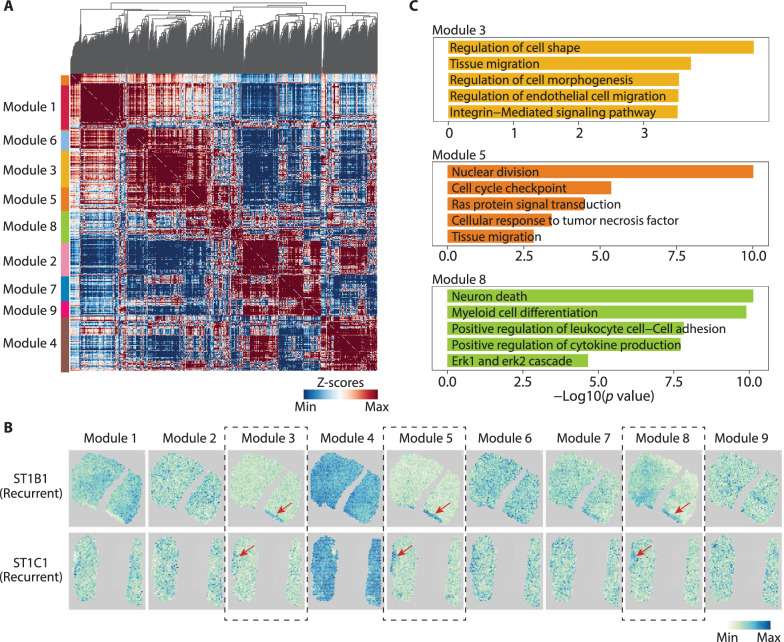
Gene signatures analysis reveals the specific location of myeloid in rGBM, **A** Heatmap showing the 9 gene modules analyzed by Hotspot. **B** Spatial transcriptomic images showing the expression scores of 9 gene modules in rGBM tissues. **C** Bar plots showing the GO terms (biological processes) enriched by genes in module #3, #5, and #8 respectively

The transcriptomic complexity of myeloid has been reported in several studies, and more expression patterns were suggested to be categorized [15, 74, 75]. To refine the expression patterns of myeloid in GBM, we identified the top 1000 significantly correlated genes and further grouped them into 9 modules by Hotspot [38] (Fig. 6A; Additional file 20: Table S15). We then visualized the expression scores of these modules on ST images, it showed that several modules were specifically expressed in C7 niche of rGBM, including modules #3, #5, and #8 (Fig. 6B). Functional analysis reveals that cell migration, ECM signaling, TNF signaling, and MAFK signaling were enriched in these modules, which appears to be functional linked to the characteristics of C7 (Fig. 6C). Thus, these findings suggest that certain subgroups of myeloid cells may have their state shaped in specific microenvironment and be involved in the ECM and invasion characteristics of C7.

## Discussion

Recent studies reported several cellular and molecular changes at GBM recurrence, such as mesenchymal transition with decreased proneuronal state, increased proportion of oligodendrocytes, and enriched ECM signatures, revealing a systematic context shift of the tumor and neighborhood [14–17, 23, 24, 59, 76]. Thus, the comprehensive characterization of rGBM tissues in comparison to pGBM is indispensable for understanding the molecular mechanism of relapse and searching specific therapeutic targets [41].

**Fig. 7:**
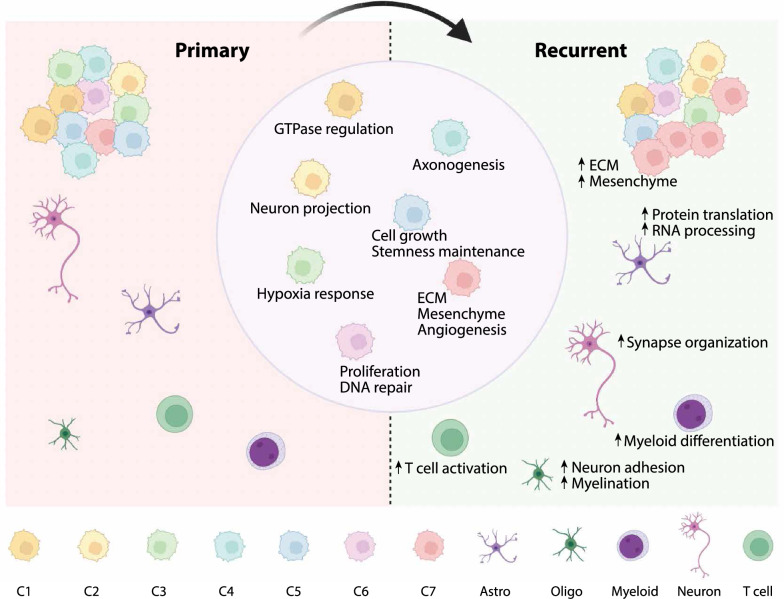
A summary model showing the transcriptional evolution at GBM recurrence

While increased expression of ECM-related genes has been linked to elevated mesenchymal-like cell state at recurrence [42], whether it is attributed to all tumor cells or certain subsets of cells is not clear yet. The latter is possible as the heterogenous nature of GBM has been reported by extensive studies, including the identification of GBM subpopulations based on the transcriptomic similarity and the GBM states based on the major expression modules. In contrast, studies of rGBM heterogeneity often refer to the gene modules deduced from pGBM [15, 24, 25], and there is still a lack of systematic understanding for the molecular heterogeneity in rGBM, in relation to pGBM and to different anatomical regions. Here, we combined tumor cells from longitudinal GBM specimens for subpopulation analysis according to the whole transcriptome, which led to the discovery of the C7 subpopulation that preferentially increased at recurrence (Fig. 7). A high score of MVP further assigned a tumor region with unique anatomic and molecular features to this cell population, which provided a reasonable explanation for the enriched angiogenesis feature of C7. Additionally, C7 is enriched with ECM-related process and shows a high score for EMT signature. As the GBM cells often infiltrate along the blood vessel and the infiltrative GBM cells are a major cause of recurrence [77–79], it is likely that C7 subpopulation is enriched in the MVP region, contributing to angiogenesis and TME remodeling via its enhanced expression of ECM component genes [80, 81]. The finding of myeloid subpopulations at the MVP region is particularly interesting to further study the interaction of tumor and immune cells that may promote MES transition and tumor progression.

Given the role of the ECM to foster tumorigenesis and relapse [82–84], its component genes have been potential targets for cancer treatment [14, 63, 85–87]. Therefore, it is essential to find key regulators for aberrant ECM production and determine the molecular features of key cell subpopulations responsible for ECM gene expression. In our study, we identified MAFK as a potential regulator by both scRNA-seq and scATAC-seq data, which might be involved in the regulation of most signature genes for rGBM. MAFK functions in several cancers, such as EMT induction and tumorigenesis in triple-negative breast cancer [54, 88]. In addition, the MAFK and MAFG deficiency has been reported to induce ECM genes misexpression in lens embryonic development [89]. Whether MAFK plays a significant role in mediating ECM regulation in rGBM is an intriguing question and deserves future investigation. In terms of regulators of the C7 subpopulation, NFATC4 was reported in several cancers [90], such as ovarian cancer [91], schwannoma [92], and hepatocellular carcinoma [71], and was suggested as the target in cancer treatment [71, 93]. At the molecular level, NFATC4 was associated with ECM production, and the NFAT family was involved in angiogenesis and metastasis [94–96]. Thus, NFATC4 might be specifically activated as an ECM regulator in the rGBM cells of the MVP region.

## Conclusions

Collectively, our in-depth single-cell and ST analyses of longitudinal GBM specimens extend our mechanistic understanding of state transition of rGBM in relation to increased ECM gene expression, identify an important subpopulation of rGBM cells and key molecular regulators. Evident gene expression alteration is also revealed in non-tumor cells, especially myeloid cells with distinct gene modules and enriched locations. With increased scale of studies targeting the mechanism of rGBM progression, more molecular candidates will be discovered and validated for future investigation in targeted therapy for rGBM.

## Supplementary Information

**Additional file 1**. **Fig. S1**. Quantification control (QC) for scRNA-seq data. **A**–**C** Violin plots for the number of genes (**A**), number of counts (**B**), and percentage of mitochondrial genes (**C**) in each sample after QC. **D**–**F** t-SNE plots labeled by sample (**D**), cluster (**E**), and malignant status inferred by inferCNV (**F**).

**Additional file 2**. **Fig. S2**. rGBM GS genes are upregulated in GBM cells at recurrence. **A** Scatter plot showing the DEGs of pGBM and rGBM cells compared with astrocytes respectively. **B** Bar plot showing the enriched GO terms (biological processes) for DEGs from Fig. S2A. The color corresponds to Fig. S2A. **C** Bar plots showing the expression of rGBM GS genes in primary and recurrent samples. **D** Scatter plot showing the regulatory activity score for deduced TFs activated in GBM cells from primary samples by RABIT [36]. **E** Venn diagram showing the intersection of deduced TFs by scRNA-seq data and differential motifs from scATAC-seq data [15]. **F** Overall survival curves of glioma patients stratified by MAFK expression using GlioVis [27].

**Additional file 3**. **Fig. S3**. Subpopulations for GBM cells across the longitudinal samples. **A** t-SNE plot showing the sample type origins of GBM cells (i.e., primary and recurrent samples). **B** t-SNE of GBM cell subpopulations split by primary and recurrent samples. **C** and **D** t-SNE plots labeled by sample (**C**) and patient (**D**). **E** Heatmap showing the expression of top 50 DEGs for each GBM cell subpopulations. **F** and **G** Bar plots showing the enriched GO terms of DEGs in GBM cell subpopulations, including C1 (**F**) and C4 (**G**). **H** and **I** t-SNE plot showing the expression scores of TNF pathway (**H**) and AP1 family (**I**) in GBM cell subpopulations. (**J**) Dot plot showing the expression of the known diagnostic markers across GBM cell subpopulations.

**Additional file 4**. **Fig. S4**. Expression of signatures depicts the characteristic of GBM cell subpopulations. **A** t-SNE feature plot showing the expression of genes from P and GS signatures. **B** SNE showing the expression of genes from Ivy anatomic features. **C** and **D** Spatial transcriptomic images showing the expression scores of C7 DEGs (**C**) and CancerSEA signatures (**D**) across ST samples. C7 cells increased in rGBM and concentrated in a niche with highly ECM, MVP, angiogenesis, EMT, and invasion scores.

**Additional file 5**. **Fig. S5**. Comparative analysis portrays the variation of non-tumor cells across longitudinal GBM samples. **A**–**E** Bar plots depicting the enriched GO terms of upregulated genes in oligodendrocyte (**A**), neuron (**B**), astrocyte (**C**), myeloid (**D**), and T cell (**E**) from recurrent samples compared with primary samples. **F** Spatial transcriptomic images showing the expression scores of two myeloid DEG signatures (the first DEG signature is from the comparison of myeloid in MES subtype to those from other subtypes, the second is from the comparison of myeloid in MES subtype to those from normal brain tissue).

**Additional  file 6**. **Table S1**. DEG list for each cell type.

**Additional file 7**. **Table S2**. DEG list for pGBM and rGBM cells compared with astrocytes respectively.

**Additional file 8**. **Table S3**. DEG list between pGBM cells and rGBM cells.

**Additional file 9**. **Table S4**. Enriched GO terms for DEGs between pGBM cells and pGBM cells.

**Additional file 10**. **Table S5**. Enriched GO terms for identified rGBM GS genes.

**Additional file 11**. **Table S6**. The inferred TFs in GBM cells from primary and recurrent samples respectively by RABIT.

**Additional file 12**. **Table S7**. The intersection of rGBM GS genes and MAFK targets from ChIP-Atlas.

**Additional file 13**. **Table S8**. The deconvolution results for bulk RNA-seq in GLASS and CGGA.

**Additional file 14**. **Table S9**. DEG list for each GBM cell subpopulation.

**Additional file 15**. **Table S10**. Signatures used in this study.

**Additional file 16**. **Table S11**. The proportion of GBM cell subpopulations labeled by several signatures.

**Additional file 17**. **Table S12**. The list for the three patterns of genes from monocle heatmap.

**Additional file 18**. **Table S13**. Enriched GO terms for each gene group identified in monocle.

**Additional file 19**. **Table S14**. Enriched GO terms for upregulated genes in each non-tumor cell type at GBM recurrence.

**Additional file 20**. **Table S15**. The expression modules of myeloid identified in GBM.

## Data Availability

The scRNA-seq data analyzed in this study were accessed from the published study (https://doi.org/10.1038/s43018-022-00475-x) and the accession code is GSE174554. The processed data in this study were accessible via an online interface (https://db.cngb.org/cdcp/visualization?project=CNP0004174). The ST data of longitudinal GBM samples were collected from the published study (https://doi.org/10.1038/s41467-023-38186-1) and accessible through Github website (https://github.com/adithyakan/reconvolving_gbm) [28]. The GLASS RNA-seq datasets were retrieved from https://www.synapse.org [17] and the CGGA RNA-seq datasets were downloaded via GlioVis data portal (http://gliovis.bioinfo.cnio.es) [26, 27]. All other data supporting the findings of this study are available from the corresponding authors on reasonable request.

## Abbreviations

GBM: Glioblastoma
scRNA-seq: Single-cell RNA-sequencing
pGBM: Primary GBM
rGBM: Recurrent GBM
GLASS: Glioma longitudinal analysis
CGGA: Chinese Glioma Genome Atlas
ST: Spatial transcriptomic
t-SNE: t-distributed stochastic neighbor embedding
GO: Gene ontology
PPI: Protein–protein interaction
rGBM GS: rGBM gene signature
OS: Overall survival
TME: Tumor microenvironment
TMZ: Temozolomide
AP-1: Activator protein 1
ECM: Extracellular matrix
CNV: Copy number variation
TFs: Transcription factors
scATAC-seq: Single-cell assay for transposase-accessible chromatin with sequencing
P cells: Proliferating cells
GS cells: Quiescent glioblastoma stem cells
CL_LE: Mixture of cellular tumor and leading edge
MVP: Microvascular proliferation
PAN: Pseudopalisading cells around necrosis
DEGs: Differential expressed genes
Ivy GAP: Ivy glioblastoma atlas project
KM: Kaplan–Meier
Astro: Astrocyte
Oligo: Oligodendrocyte
