# Supplementary figures and images for: Decoding key cell sub-populations and molecular alterations in glioblastoma at recurrence by single-cell analysis
Source: Acta Neuropathol Commun. 2023 Jul 31;11:125. doi: 10.1186/s40478-023-01613-x (PMC10391841; doi:10.1186/s40478-023-01613-x)

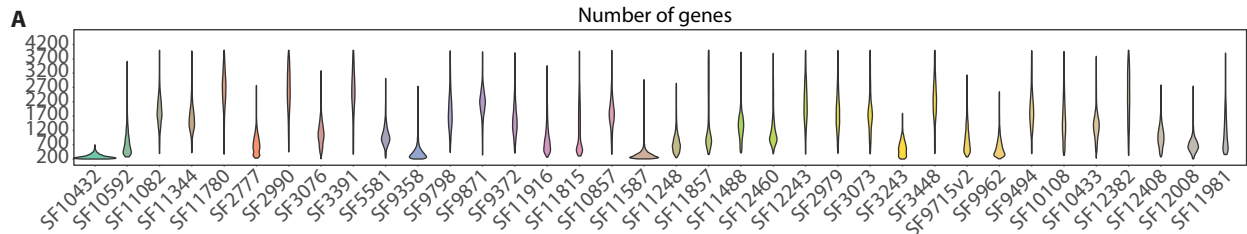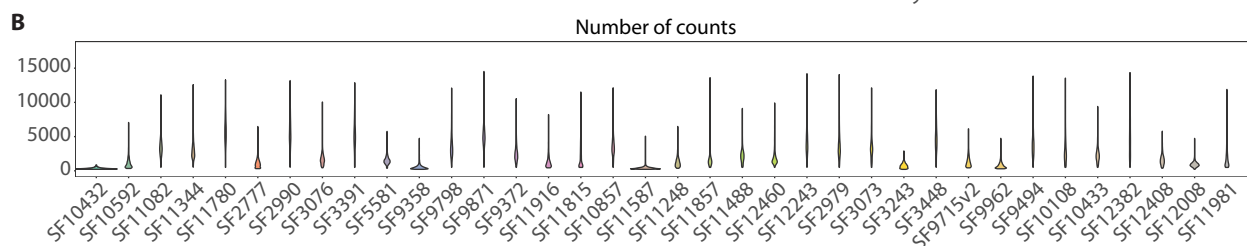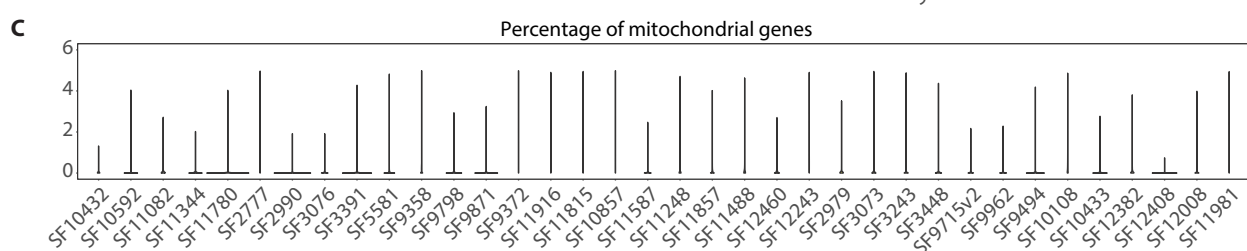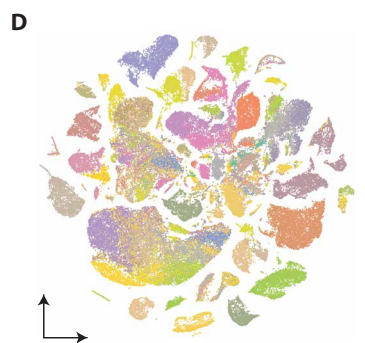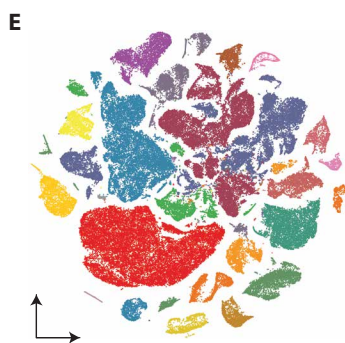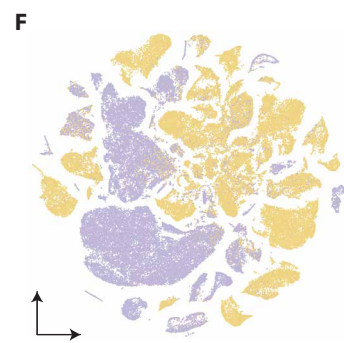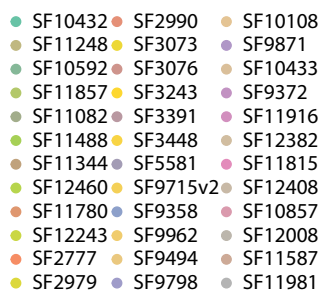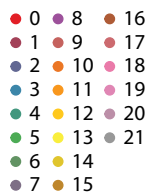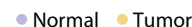

Supplement: Supplementary file 1 — Additional file 1. Fig. S1. Quantification control (QC) for scRNA-seq data. A–C Violin plots for the number of genes (A), number of counts (B), and percentage of mitochondrial genes (C) in each sample after QC. D–F t-SNE plots labeled by sample (D), cluster (E), and malignant status inferred by inferCNV (F). [file 40478_2023_1613_MOESM1_ESM.pdf]

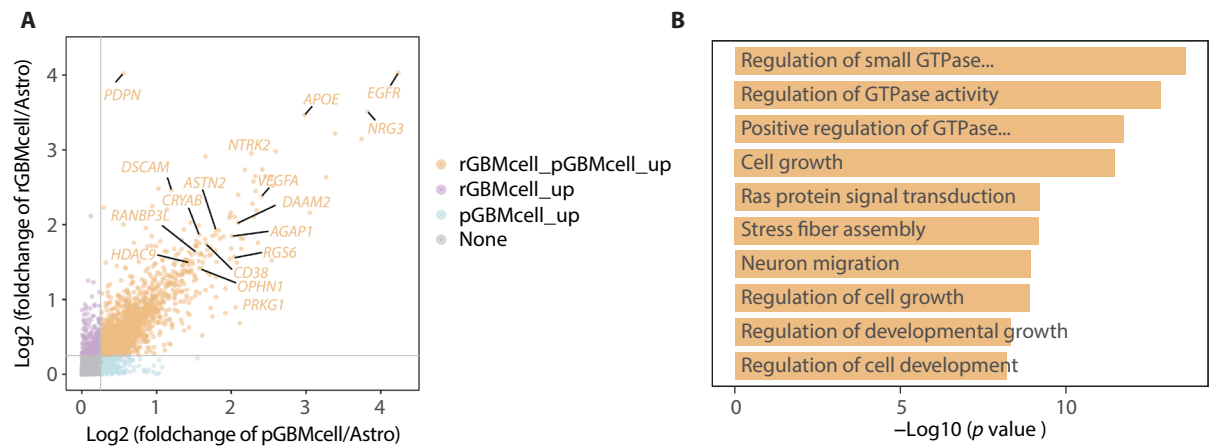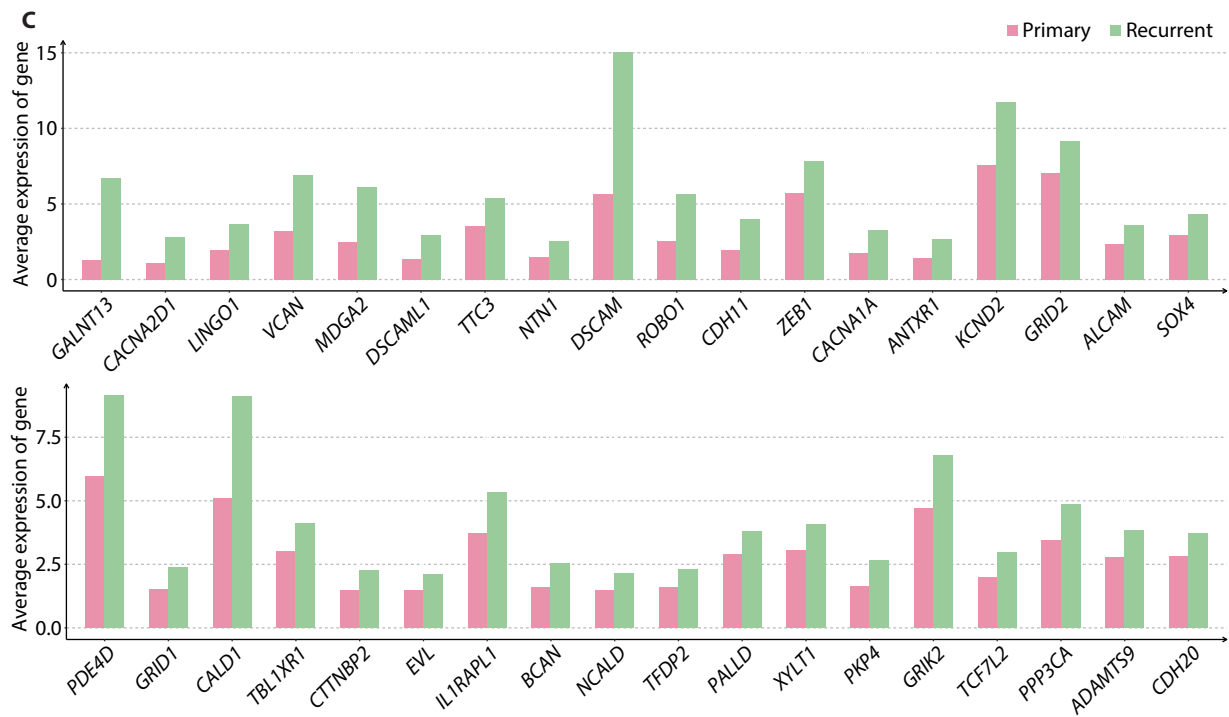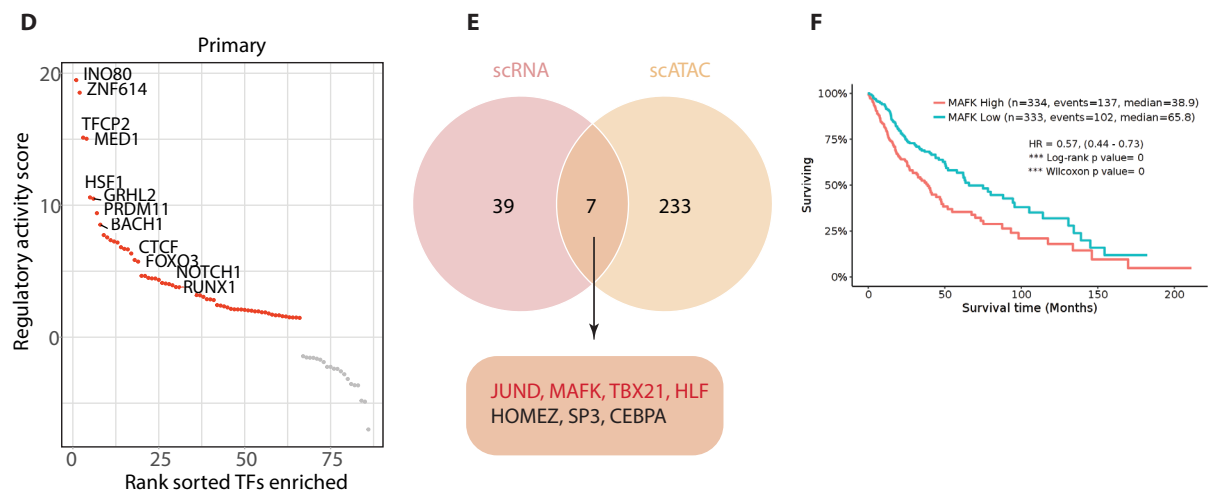

Supplement: Supplementary file 2 — Additional file 2. Fig. S2. rGBM GS genes are upregulated in GBM cells at recurrence. A Scatter plot showing the DEGs of pGBM and rGBM cells compared with astrocytes respectively. B Bar plot showing the enriched GO terms (biological processes) for DEGs from Fig. S2A. The color corresponds to Fig. S2A. C Bar plots showing the expression of rGBM GS genes in primary and recurrent samples. D Scatter plot showing the regulatory activity score for deduced TFs activated in GBM cells from primary samples by RABIT [36]. E Venn diagram showing the intersection of deduced TFs by scRNA-seq data and differential motifs from scATAC-seq data [15]. F Overall survival curves of glioma patients stratified by MAFK expression using GlioVis [27]. [file 40478_2023_1613_MOESM2_ESM.pdf]

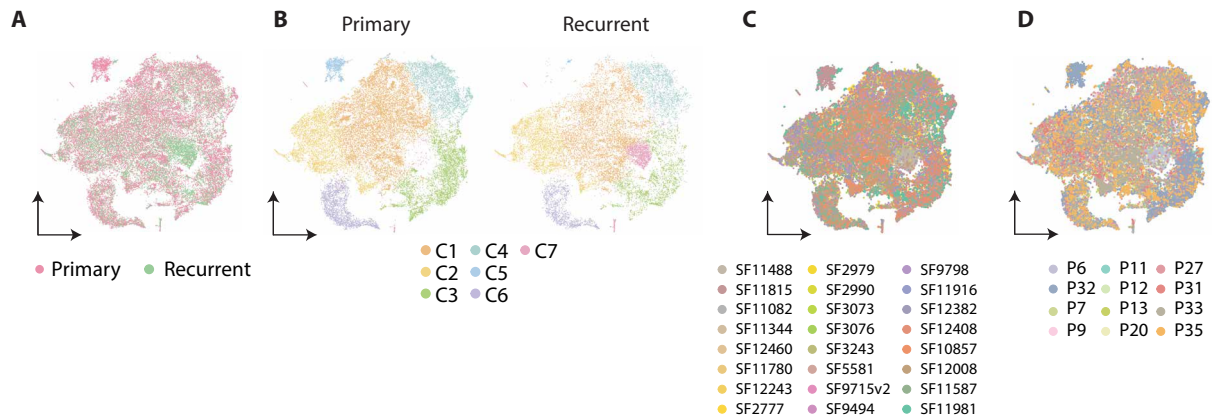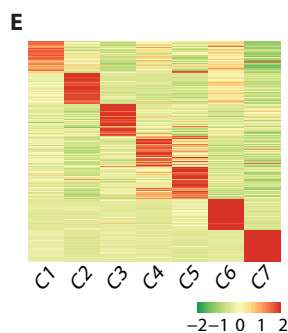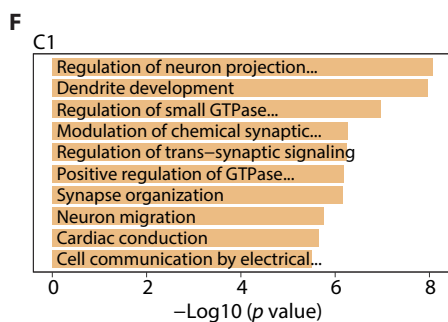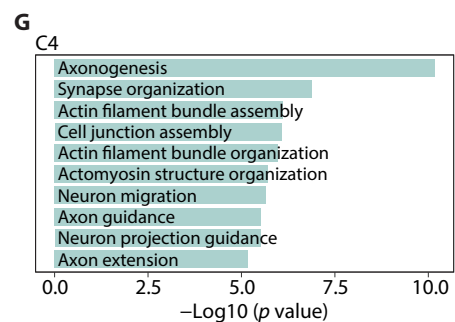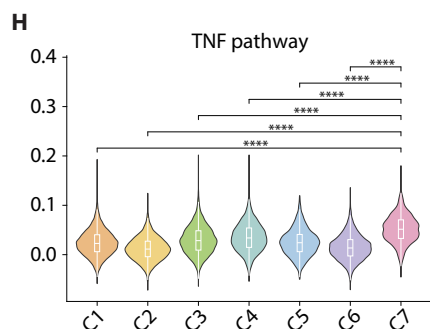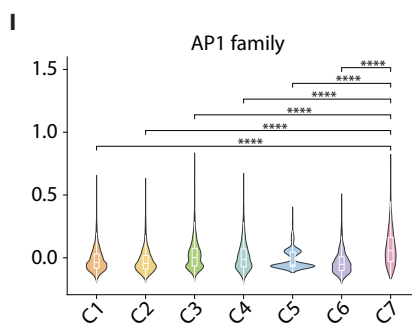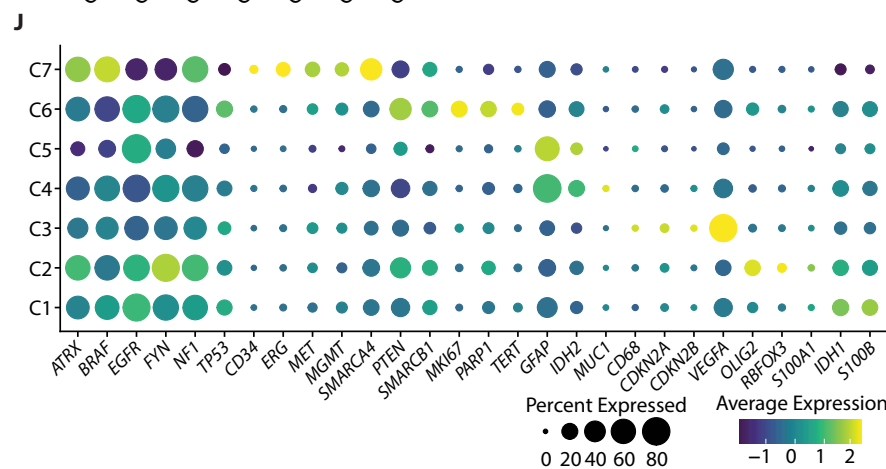

Supplement: Supplementary file 3 — Additional file 3. Fig. S3. Subpopulations for GBM cells across the longitudinal samples. A t-SNE plot showing the sample type origins of GBM cells (i.e., primary and recurrent samples). B t-SNE of GBM cell subpopulations split by primary and recurrent samples. C and D t-SNE plots labeled by sample (C) and patient (D). E Heatmap showing the expression of top 50 DEGs for each GBM cell subpopulations. F and G Bar plots showing the enriched GO terms of DEGs in GBM cell subpopulations, including C1 (F) and C4 (G). H and I t-SNE plot showing the expression scores of TNF pathway (H) and AP1 family (I) in GBM cell subpopulations. (J) Dot plot showing the expression of the known diagnostic markers across GBM cell subpopulations. [file 40478_2023_1613_MOESM3_ESM.pdf]

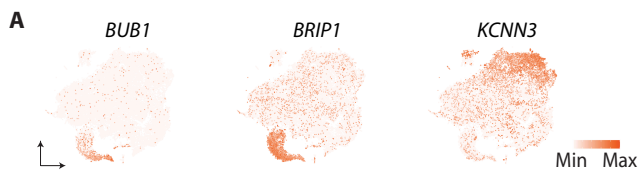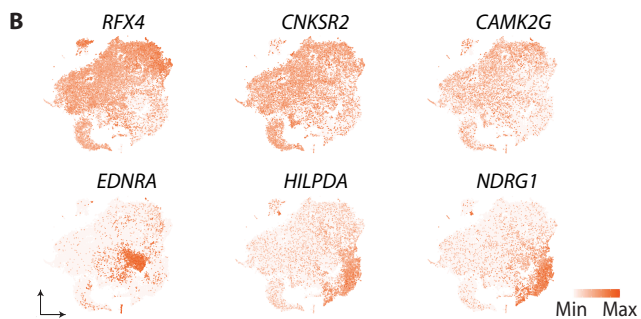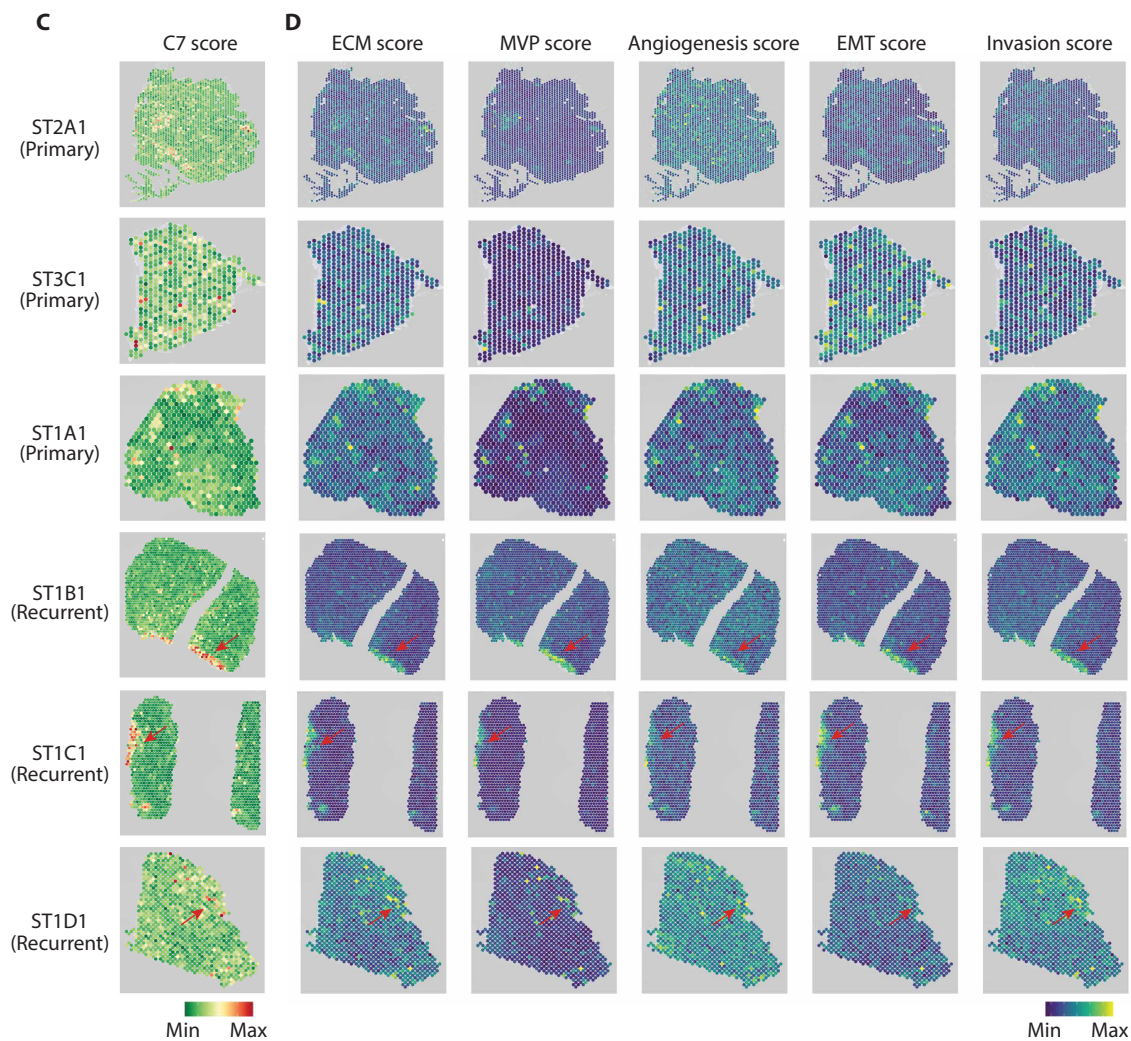

Supplement: Supplementary file 4 — Additional file 4. Fig. S4. Expression of signatures depicts the characteristic of GBM cell subpopulations. A t-SNE feature plot showing the expression of genes from P and GS signatures. B SNE showing the expression of genes from Ivy anatomic features. C and D Spatial transcriptomic images showing the expression scores of C7 DEGs (C) and CancerSEA signatures (D) across ST samples. C7 cells increased in rGBM and concentrated in a niche with highly ECM, MVP, angiogenesis, EMT, and invasion scores. [file 40478_2023_1613_MOESM4_ESM.pdf]

**A** Oligodendrocyte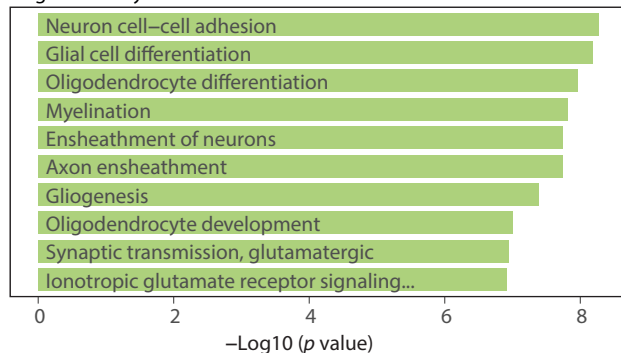**B**

## Neuron

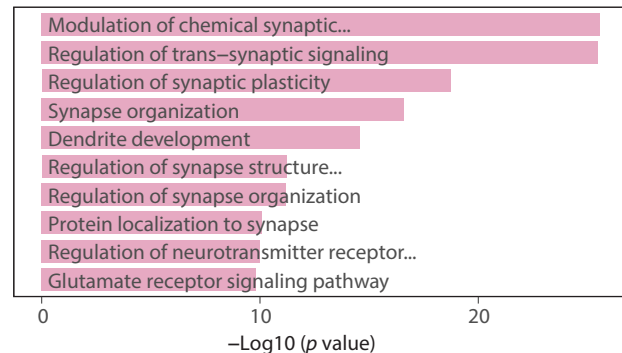**C**

## Astrocyte

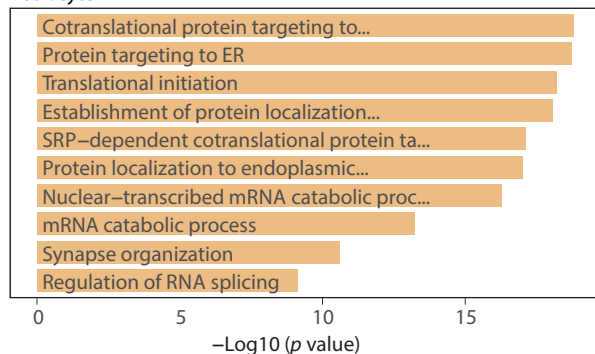**D**

## Myeloid

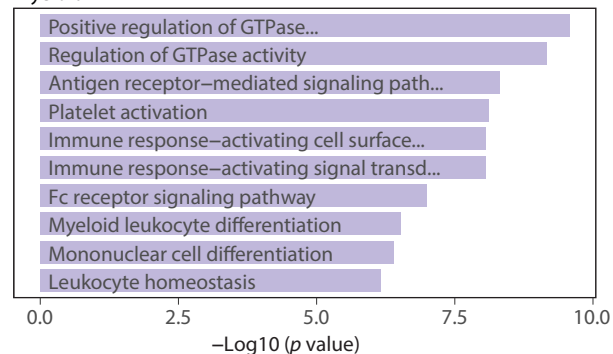**E**

## T cell

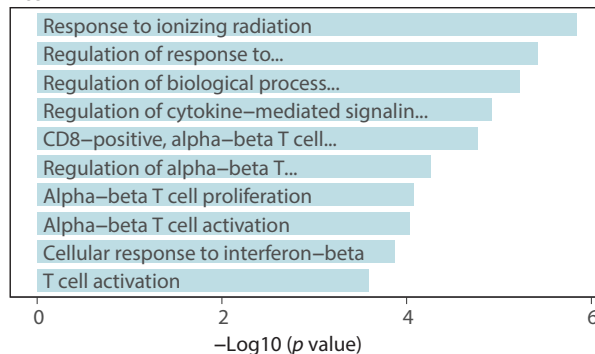**F**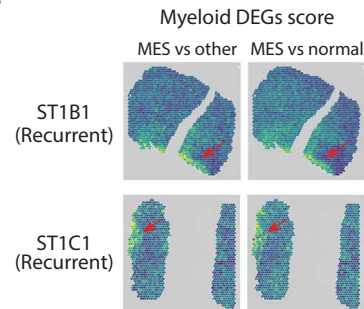

Supplement: Supplementary file 5 — Additional file 5. Fig. S5. Comparative analysis portrays the variation of non-tumor cells across longitudinal GBM samples. A–E Bar plots depicting the enriched GO terms of upregulated genes in oligodendrocyte (A), neuron (B), astrocyte (C), myeloid (D), and T cell (E) from recurrent samples compared with primary samples. F Spatial transcriptomic images showing the expression scores of two myeloid DEG signatures (the first DEG signature is from the comparison of myeloid in MES subtype to those from other subtypes, the second is from the comparison of myeloid in MES subtype to those from normal brain tissue). [file 40478_2023_1613_MOESM5_ESM.pdf]
